# Mapping the Inorganic and Proteomic Differences among Different Types of Human Teeth: A Preliminary Compositional Insight

**DOI:** 10.3390/biom10111540

**Published:** 2020-11-11

**Authors:** Vaibhav Sharma, Simran Rastogi, Kaushal Kumar Bhati, Alagiri Srinivasan, Ajoy Roychoudhury, Fredrik Nikolajeff, Saroj Kumar

**Affiliations:** 1 Departement of Biophysics, All India Institute of Medical Sciences, New Delhi 110029, India; sr_1997du@yahoo.com; 2 Louvain Institute of Biomolecular Science, Université Catholique de Louvain, 1348 Ottignies-Louvain-la-Neuve, Belgium; kaushalkbhati@gmail.com; 3 Department of Biochemistry, Jamia Hamdard University, New Delhi 110062, India; srinivasanalagiri@gmail.com; 4 Centre for Dental Education and Research (CDER), All India Institute of Medical Sciences, New Delhi 110029, India; ajoyroy@hotmail.com; 5 Department of Health Sciences, Lulea University of Technology, 97187 Lulea, Sweden; Fredrik.Nikolajeff@angstrom.uu.se

**Keywords:** tooth proteome, mass spectrometry, teeth composition, inductively coupled plasma resonance mass spectrometry, protein-protein interactions

## Abstract

In recent years, studies on mineralized tissues are becoming increasingly popular not only due to the diverse mechanophysical properties of such materials but also because of the growing need to understand the intricate mechanism involved in their assembly and formation. The biochemical mechanism that results in the formation of such hierarchical structures through a well-coordinated accumulation of inorganic and organic components is termed biomineralization. Some prime examples of such tissues in the human body are teeth and bones. Our current study is an attempt to dissect the compositional details of the inorganic and organic components in four major types of human teeth using mass spectrometry-based approaches. We quantified inorganic materials using inductively coupled plasma resonance mass spectrometry (ICP-MS). Differential level of ten different elements, Iron (Fe), Cadmium (Cd), Potassium (K), Sulphur (S), Cobalt (Co), Magnesium (Mg), Manganese (Mn), Zinc (Zn), Aluminum (Al), and Copper (Cu) were quantified across different teeth types. The qualitative and quantitative details of their respective proteomic milieu revealed compositional differences. We found 152 proteins in total tooth protein extract. Differential abundance of proteins in different teeth types were also noted. Further, we were able to find out some significant protein-protein interaction (PPI) backbone through the STRING database. Since this is the first study analyzing the differential details of inorganic and organic counterparts within teeth, this report will pave new directions to the compositional understanding and development of novel in-vitro repair strategies for such biological materials.

## 1. Introduction

Millions of years of evolution have resulted in numerous natural materials with a high degree of sophistication which are associated with many disciplines of science including chemistry, material science, physics, biology, and engineering to name a few. The ingenious construction of biological hard tissues has always fascinated the scientific community. Unlike soft tissues, hard tissues are highly mineralized. Notable examples in the human body are bone, teeth, and calcitic otoconia of the internal ear. These biological composites contain an inorganic phase (minerals) and organic (primarily proteins) components that orchestrates the formation of such hierarchical structures through the process of biomineralization [1,2]. Investigations on hard tissues have always been a daunting task due to plenteous reasons which include getting rid of excess salts, low protein yield, and protein heterogeneity issues due to the presence of diverse post-translational modifications profile within these proteins [3].

Human teeth consist of three layers of mineralized tissue namely the outermost enamel, dentin forms the middle layer, and the inmost layer is called the cementum. Overall, 80% of the teeth structure constitutes of the inorganic part which is hydroxyapatite (HA), a form of calcium phosphate that is also known to be present in bones. The remaining fraction contains a small number of growth factors, lipids, and primarily proteins, most of which are known to be intrinsically disordered [4,5]. On the inorganic end, HA serves as the key mineral constituent, in addition to different elements that are present in minute quantities. The “chemical signatures” of HA have already been discussed in detail through an ample number of studies from enamel [6,7,8], dentine [9], and the cementum [10] in addition to complete human teeth [11,12], largely through Fourier transform infrared spectroscopy (FTIR). The details of elemental composition within teeth are important as they are known to be present in tooth enamel [13]. The presence of even minuscule concentrations of such elements has been reported to influence the size and organization of apatite crystals, which in turn has an impact on the hardness of the enamel [14]. Also, different trace elements have been linked to various roles like caries protective and caries promotion [15,16]. In practice, atomic absorption spectroscopy (AAS) [17] and inductively coupled plasma resonance mass spectrometry (ICP-MS) [18,19] could be used to quantitate the elemental composition of biomaterials like teeth.

Besides these aforesaid inorganic players, protein molecules are known to be the key influencers of tooth development [20], biomineralization [1,21,22], and can also aid in regeneration potentials [23,24], despite this fact not many proteomic studies have been attempted in the past. Proteomic studies from individual tooth tissues like dentin [25,26] and pulp [27] are reported in the past. In the case of whole human teeth, only a single study has shown the EDTA solubilized proteins from a complete tooth and reported their tooth regenerative capacities through cell line-based assays [28].

Our previous report has already highlighted the preliminary differences within these different tooth protein extracts through gel-based assays [1]. In this current study, we examined the differences in four major classes of human teeth concerning their inorganic and organic constitutions. Organizing such a study was a daunting task in terms of technical challenges we need to overcome. Still, we are essentially rooting on the importance of this work since mapping such differential changes would reveal the compositional details of different teeth types and may aid in understanding the variables that determine different shapes and functionalities. Moreover, this study would also be useful for designing synthetic approaches for efficient peptide-based mineralization of teeth.

**Figure 1:**
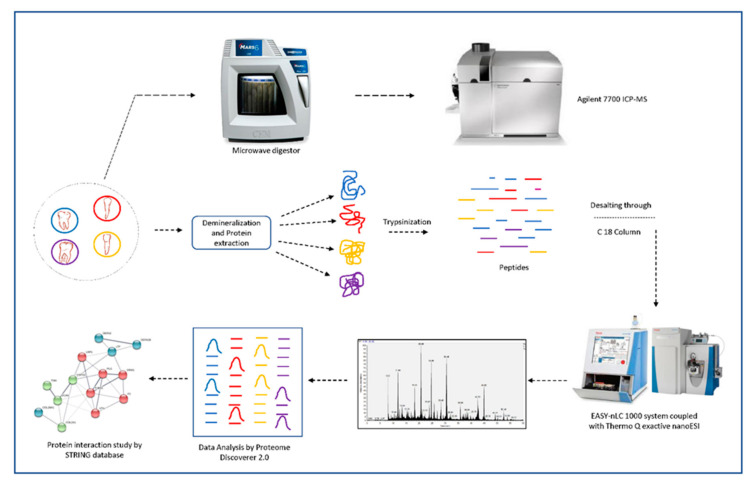
Schematic illustration to highlight the process of mineral and organic molecules identification within different teeth through ICP-MS and mass spectrometry-based methodologies.

The overall approach we followed is depicted as a scheme in Figure 1.

## 2. Materials and Methods

### 2.1. Collection of Human Teeth and Isolation of Tooth Proteins

This study was approved by the Institute Ethics Committee for Post Graduate Research of All India Institute of Medical Sciences (Ref no. IECPG-387/29.06.2016). All experiments were performed in accordance with relevant guidelines and regulations. Informed consent was received from all the participants. Samples were collected from the Center for Dental Education and Research (CDER), AIIMS, New Delhi. The adults were aged 22–25 years and removal of teeth were due to orthodontic reasons. Collected teeth were lyophilized and kept in liquid nitrogen until used. Teeth were washed thoroughly with deionized water (2–3 times). After washing, teeth of similar types were pooled together and crushed in a pestle and mortar in the presence of liquid nitrogen until a fine powder was formed. For the isolation of protein from different teeth, a protocol standardized by Sharma et al. was followed [1]. Briefly, 10 g of the powder was taken and mixed with 20 mL of demineralization solution containing 0.1 M EDTA, 100 mM NaCl, and protease inhibitors (Roche) at 20 °C for 2 days, centrifuged at 10,000 rpm, and collected supernatant was dialyzed against HEPES buffer, pH 7.4. The clarified supernatant was further concentrated by the membrane-based cut off filters (3.5 kDa), which helps to remove impurities and salts as well as also reduced the loss of smaller proteins from our extracts. The protein concentration was measured by BCA kit (Pierce, Thermo Scientific, Rockford, IL, USA) according to the manufacturer’s instructions and protein extracts were checked by running an SDS-PAGE gel.

### 2.2. Inductively Coupled Plasma Resonance Mass Spectroscopy (ICP-MS)

Prior to analysis, teeth samples were washed with ICP-MS grade water and speed frost dried overnight. The equal weight of competitive samples was microwave digested in HNO3 (Suprapur^®^ Merck, Darmstadt, Germany) using MARS^TM^ Microwave tissue digestion system. These digested samples were further diluted up to 50 mL in ICP-MS grade water and prepared for ICP-MS run in triplicates. The total metal analysis was performed by inductively coupled plasma mass spectrometry (ICP-MS; 7700×Agilent Technologies, Santa Clara, CA, USA). The ppb values we obtained after ICP-MS analysis were normalized according to initial sample weight and dilutions factors as given in the following formula:

(1) $$Metal\;\left(\frac{\mu g}{g\;dry\;weight\;of\;the\;sample}\right)=\frac{ICP-MS\;reading\;\left(ppb\right)\times \left(\frac{Dilution}{weight}\right)}{1000}$$

The values shown in the graphs are an average of three independent experiments and the standard deviation plotted as error bars.

### 2.3. Label-Free Quantification of Tooth Proteins through Mass Spectrometry

Sample Preparation—Samples were taken and reduced with 5 mM tris 2-carboxyethyl phosphine (TCEP) and further alkylated with 50 mM iodoacetamide and then digested with Trypsin (1:50, Trypsin/lysate ratio) for 16 h at 37 °C. Digested samples were cleaned using a C18 silica cartridge to remove the salt and dried using a speed vac. The dried pellet was resuspended in buffer A (5% acetonitrile, 0.1% formic acid).

Mass Spectrometric Analysis of Peptide Mixtures—All the experiments were performed using EASY-nLC 1000 system (ThermoFisher Scientific, Illinois, USA) coupled to Thermo Fisher-QExactive equipped with nanoelectrospray ion source. 1.0 μg of the peptide mixture was resolved using 25 cm PicoFrit column (360 μm outer diameter, 75 μm inner diameter, 10 μm tip) filled with 1.8 μm of C18-resin (Dr. Maisch Ammerbuch, Germany). The peptides were loaded with buffer A and eluted with a 0–40% gradient of buffer B (95% acetonitrile, 0.1% formic acid) at a flow rate of 300 nL/min for 90 min. MS data were acquired using a data-dependent top10 method dynamically choosing the most abundant precursor ions from the survey scan. We performed three technical replicates in this experiment. Since we have used proteins from pooled teeth samples of different individuals, we did not run biological replicates for this study.

Data Processing—All samples were processed, and 4 RAW files generated were analyzed with Proteome Discoverer (v2.2) against the Uniprot Human reference proteome database. For the Sequest search, the precursor and fragment mass tolerances were set at 10 ppm and 0.5 Da, respectively. The protease used to generate peptides, i.e., enzyme specificity was set for trypsin/P (cleavage at the C terminus of “K/R: unless followed by “P”) along with a maximum missed cleavages value of two. Carbamidomethyl on cysteine as fixed modification and oxidation of methionine and N-terminal acetylation were considered as variable modifications for database search. Both peptide spectrum match and protein false discovery rate were set to 0.01 FDR. Heat maps, correlation plot, and principal component analysis (PCA) were prepared through R programming.

### 2.4. Protein Interaction Studies

The search tool for retrieval of interacting genes (STRING)(https://string-db.org/) database which integrates known and predicted protein-protein interactions (PPIs) can be applied to decipher the functional interactions of proteins [29]. To seek potential interactions among tooth proteins, the STRING (Version 11.0) tool was employed. Active interaction sources including text mining, experiments, databases, and co-expression as well as species limited to “Homo sapiens” with the confidence of 0.4 was set. Clustering was performed by k mean clustering. In the networks, the nodes correspond to the proteins and the edges represent the interactions. The thickness of edges represents the interaction strength.

## 3. Results and Discussion

### 3.1. The Inorganic Analysis Revealed Differences in Teeth Mineral Composition

Trace elements can enter our body through multiple routes such as food, water, or exposure to the environment [30,31,32] and can get incorporated into enamel/dentin crystals. Although their presence is confirmed within teeth, not much literature is available to comment upon their functional significance at the level of teeth mineralization or morphology. In this study, a total of 10 elements were quantified among different teeth of humans.

**Figure 2:**
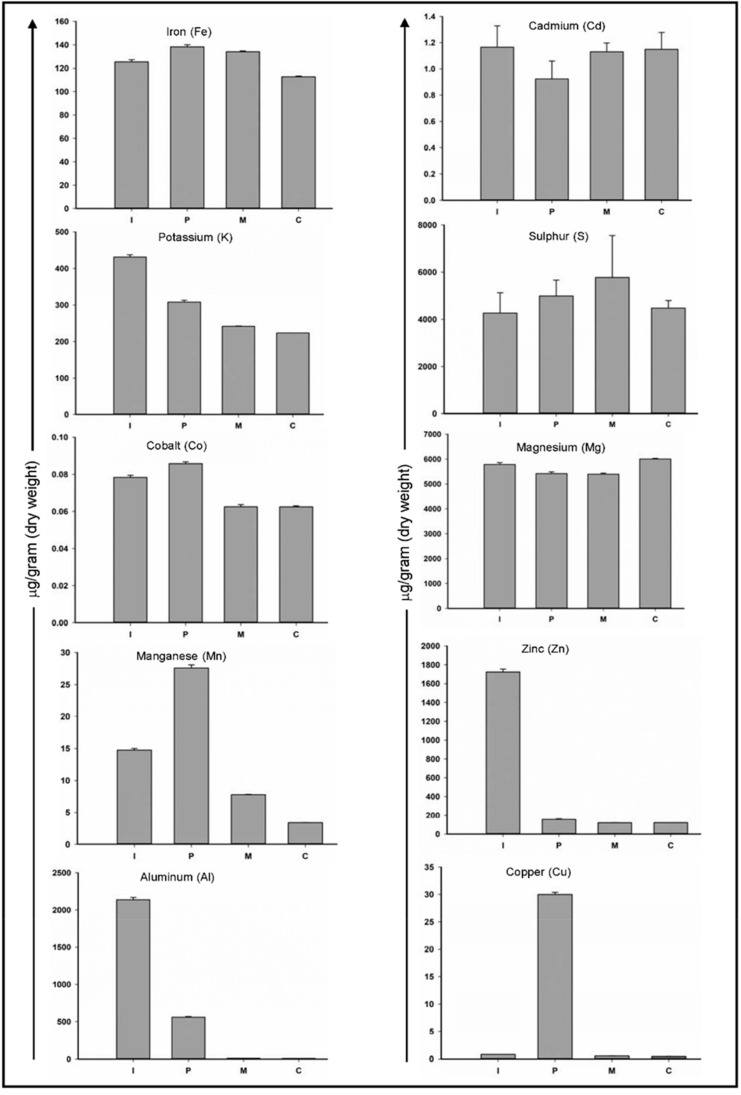
Comparative ICP-MS profile of four different types of teeth. Ten different trace elements were quantified. The vertical axis shows the amount of trace elements in micrograms/gram dry weight and on the horizontal axis are different teeth types where I: incisor; P: premolar; M: molar and C: canine teeth respectively. The standard deviation of three independent experiments was plotted as error bars.

As shown in Figure 2, among all elements, iron (Fe), cobalt (Co), cadmium (Cd) and magnesium (Mg) values show the least variations among four types of teeth and the value varies from 112–125 μg/g, 0.062–0.075 μg/g, 0.9–1.16 μg/g and 5400–6000 μg/g respectively. Iron is considered as an essential element of human life. It can enter the human body through the consumption of vegetables and was reported to exist in teeth [33]. It is testified that incorporation of Fe results in lowering the content of carbonate type A apatite [14]. On the other hand, cobalt and cadmium are toxic metals that can enter the body through contaminated air, water, and food. Co and Cd can easily affect teeth by replacing Ca^2+^ ions within the apatite crystal [34,35]. Magnesium is one of the most abundant trace elements in saliva and since teeth remain bathing with salivary components. Magnesium is also the most abundant trace element in enamel [36,37]. Our study also echoed with these previous findings and Mg was indeed present in the highest concentrations of 5400–6000 μg/g.

Elements such as aluminum (Al), copper (Cu), and zinc (Zn) were among the ones with the most variations within different teeth types. Within a human body, aluminum concentration increases with age and higher amounts may cause brain and skeleton disorders [38,39]. Ghadimi et al. [14] reported a negative correlation of Al concentrations with the crack length i.e., Teeth with higher concentration are resistant to longer cracks and vice versa. In our study, we observed higher concentrations of Al in incisor and premolar teeth, this is justifiable as these teeth types are less prone to cracks in comparison to molar teeth. Zinc is an essential trace element present throughout the body. In the mouth, it is naturally present in plaque, saliva, and enamel. Zinc is acquired by hydroxyapatite and competes for Ca^2+^ ions within the lattice [17]. It has a role in the remineralization of enamel and increased concentration may reduce susceptibility for dental caries [40]. In our study, we are reporting for the first time, maximum concentrations of Zn in incisor teeth, which may have a role in making its caries resistive.

One of the major limiting factors while studying the trace elements is the presence of a myriad of factors that may influence the content of heavy metals in hard tooth tissues. In addition to this, the effects of diet during pre-eruptive and post-eruptive phases also influence the results and are not clearly separated in most experimental designs, which in turn complicates the interpretation of the results. Nevertheless, the importance of trace elements in modulating tooth mineralization, development, and role in carries cannot be understated, though further studies are needed to look into the detailed role of the presence of these elements within hard tissues.

### 3.2. Proteins in Four Major Teeth Types Differ Qualitatively and Quantitatively

**Figure 3:**
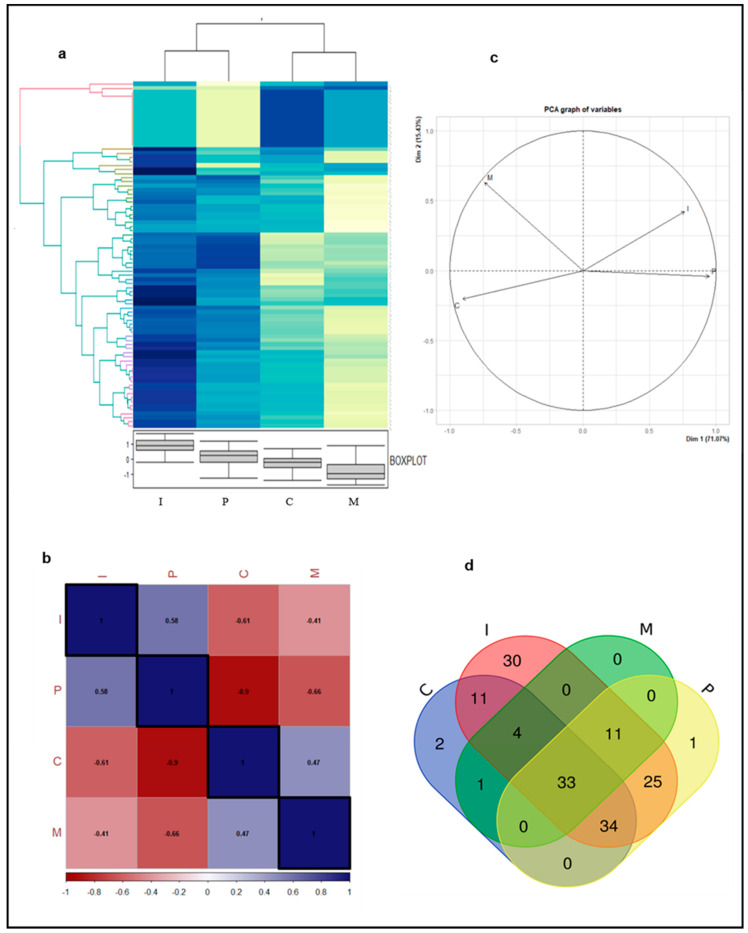
Comparative analysis of differential proteome within four types of human teeth. (**a**) Levels of protein abundance are depicted through (**a**) heatmap; (**b**) correlation plot; (**c**) principal component analysis (PCA); (**d**) Venn diagram. The different protein extracts notation is as follows I: incisor; P: premolar; M: molar and C: canine.

For proteome analysis, the tooth protein extracts were subjected to label-free quantification using mass spectrometry. A total of 152 proteins were identified (Table S1). These proteins represent different predominant biological and molecular functions as assessed through Gene Ontology (GO) analysis (Table 1). These proteins were found to be associated with various biological processes (cell differentiation, cell organization, and biogenesis, metabolic process, transport, etc.) and had diverse molecular functions (DNA binding, protein binding, enzyme regulator activity, structural molecular activity). In this study, we found Serotransferrin, fibrillin-1, components of immunoglobins, and collagen subtypes were among the most abundant proteins in terms of peptide hits. Serotransferrin is a glycoprotein known to be present in enamel pellicle and has a role in tooth morphogenesis [41,42] whereas, fibrillin-1 and collagens are extracellular matrix proteins. Collagen is the most abundant protein in dentin and also present in bone [5]. Fibrillins are cysteine-rich glycoproteins which are responsible for providing protein structural support and helps teeth to withstand compression forces during mastication [43]. In addition to collagens, several non-collagenous proteins like Versican, Vitronectin, Hyaluronan, and proteoglycan link protein were also found in these protein extracts. Several other proteins reported to have a role in tooth development and odontogenesis were also enriched in our list, these include Tenascin [44,45], Thrombospondin-1 [46], Laminins [47,48], Fibronectin [49], Dickkopf related-protein 3 [50], and Vitronectin [51]. Furthermore, proteins like antileukoproteinase, lactotransferrin, and serotransferrin which are detected in our extracts are known to be present in saliva and which are adsorbed in the associated enamel pellicle. This acquired enamel pellicle from saliva plays a crucial role in crystal growth homeostasis of teeth and in modulating the process of mineralization [52,53]. A comparative analysis was done through label-free quantification and a relative abundance of all proteins was calculated. Interestingly, differential change in protein abundance could be seen among different teeth types we investigated (Figure 3a). Incisor and molar teeth proteins possess the most distinct protein profiles. Most of the proteins which are highly abundant in the incisor protein extract are showing low abundance levels in the molar teeth extract. A similar trend can be observed in incisor vs canine and premolar vs molar protein extracts whereas there is some amount of similarities between the protein abundance profiles of incisor and premolar. Here a noteworthy point is the use of accurate terminology, these abundance changes should not be considered as an actual expression profile due to the presence of different protein species/isoforms that may result from a variety of posttranslational modifications, protein splicing, degradation, or emigration [54,55]. These tooth extracts were further analyzed through a correlation plot and principal component analysis (PCA). In accordance with the heat map and the correlation plot, PCA analysis also showed that the protein composition of incisor and premolar teeth are closer to each other whereas the proteins of incisor and canine or incisor and molar are distantly correlated in these models (Figure 3b,c). Since this is the first-ever report on the differential proteome profile among these four teeth types, it is difficult to explain these changes in detail, and hence further studies are needed to draw valid conclusions.

The unique and common proteins among different categories are depicted in the form of a Venn diagram (Figure 3d). We found 33 common proteins present in all four types of teeth extracts. Some of the key proteins in this list include Serotransferrin, Collagen subtypes, Tenascin, Fibrillin, Histone subtypes, and a 60kDa heat shock protein and others described in Table 1 along with their UniProt IDs. A detailed list containing the complete set of unique and common protein within all four categories is provided in Table S2.

**Figure 4:**
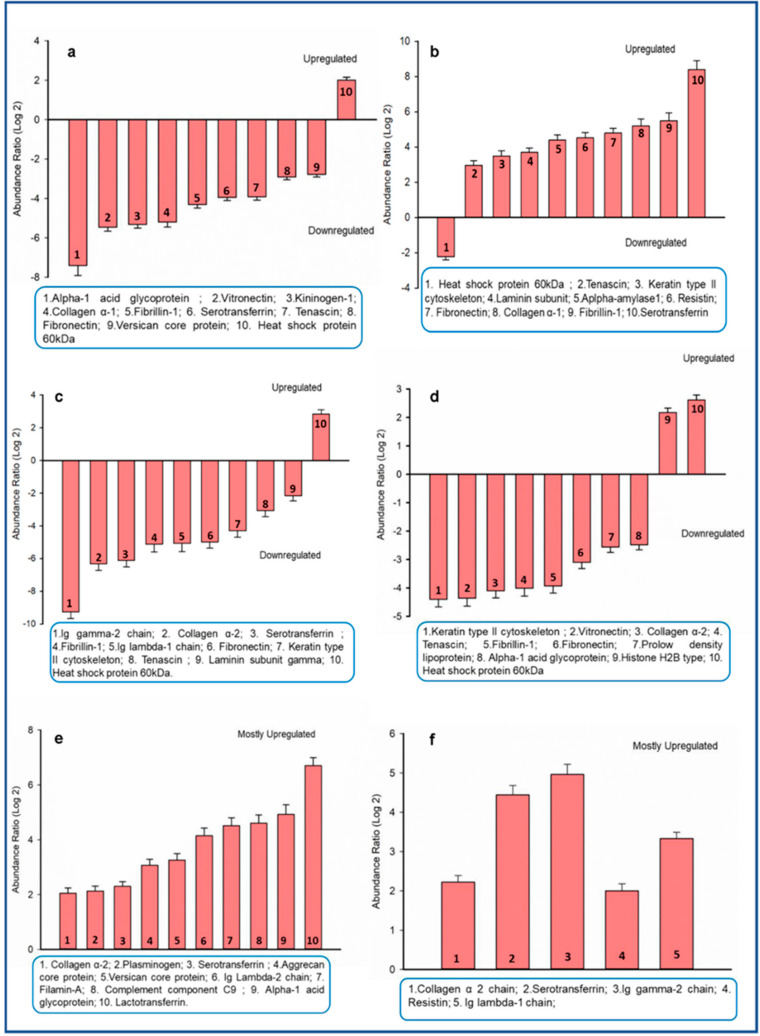
Comparative abundance profile of some key proteins found within different teeth combinations. Only Log2 abundance values more than ±2 were taken. The box below each histogram shows key proteins that are high or low in their abundance. The different combinations of proteins are as follows: (**a**) canine vs incisor; (**b**) incisor vs molar; (**c**) molar vs premolar; (**d**) canine vs premolar; (**e**) incisor vs premolar and (**f**) canine vs molar. The error bar denotes the error from three independent technical replicates.

The differential abundance was also quantitated by comparing the log 2 abundance ratios between all four teeth proteins. Some key proteins that showed high or low abundance in each of these six categories: canine vs incisor, incisor vs molar, molar vs premolar, canine vs premolar, incisor vs premolar, canine vs molar are shown (Figure 4a–f). The log 2 abundance of equal to or greater than ±2 were taken into account for the sake of representation. However, a detailed table showing a complete list of differentially abundant proteins with their respective abundance ratios (log 2 values) among the possible six combinations of different teeth protein extracts are provided in Table S3 (Tables 1–6). Many proteins exhibiting varied abundance are reported to have a role in tooth development.

### 3.3. Protein Interaction Network Studies

**Figure 5:**
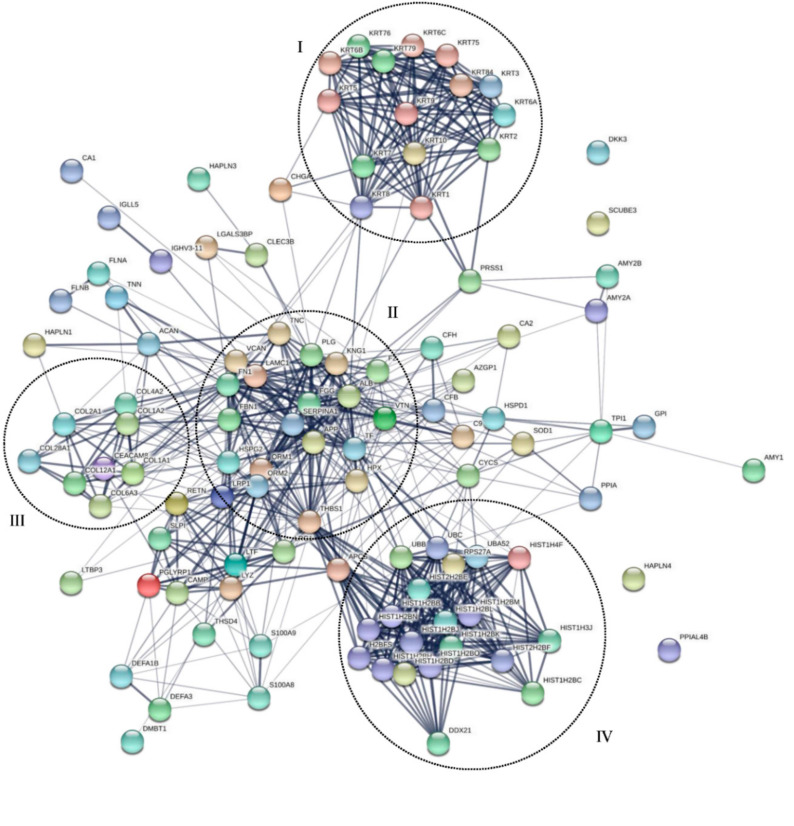
The protein-protein interaction network of tooth proteins. Nodes represent proteins and edges represent the protein-protein associations. Upon k-mean clustering, four main clusters were visible, and these are encircled for better representation (Cluster I, II, III, IV).

We used the MS data for prediction and validation by in silico protein-protein analysis. The protein-protein interactions (PPI) were assessed through the STRING tool. The cluster analysis of the complete list of tooth proteins obtained in our study resulted in four significant clusters comprising of four different class of identified proteins (Figure 5).

Cluster I included different keratins that are involved in self oligomerization and interaction with other proteins in cluster II like Fibronectin (FN1), Kininogen-1 (KNG1), and Plasminogen (PNG). Though it can be argued that sometimes keratin comes as a contaminant protein in mass-based experiments, but in this case, keratins are previously reported in tooth proteins and serve as a part of enamel organic matrix proteins [56,57]. Cluster II was found enriched in core teeth proteins like Tenascin (TNC), Fibronectin (FN1), Plasminogen (PLG), Vitronectin (VTN), Versican core protein (VCAN) and Alpha-1-acid glycoprotein (ORM-1), etc. The role of these key proteins has already been extensively described in the proteome analysis section of this manuscript. The proteins in cluster II are linked with cluster III through basement membrane heparan sulfate (HSPG2), Versican core protein (VCAN), and Fibrillin-1 (FBN1). Cluster II is primarily composed of structural proteins i.e., collagens. Collagens are the main components of the organic matrix of teeth [58,59], especially dentin and enamel. Cluster IV constitutes mainly of secretory proteins like Histones subtypes and ubiquitin variants. Various histone deacetylases have been reported to possess a prime role in tooth development and mineralization [60,61]. Since histones perform their function through gene regulation, their direct impact on protein interactions is difficult to discuss here and is beyond the scope of the present study. The proteins in cluster IV are strongly interacting and dependent on Thrombospondin-1 (THBS-1) for further interconnections with clusters II and III.

**Figure 6:**
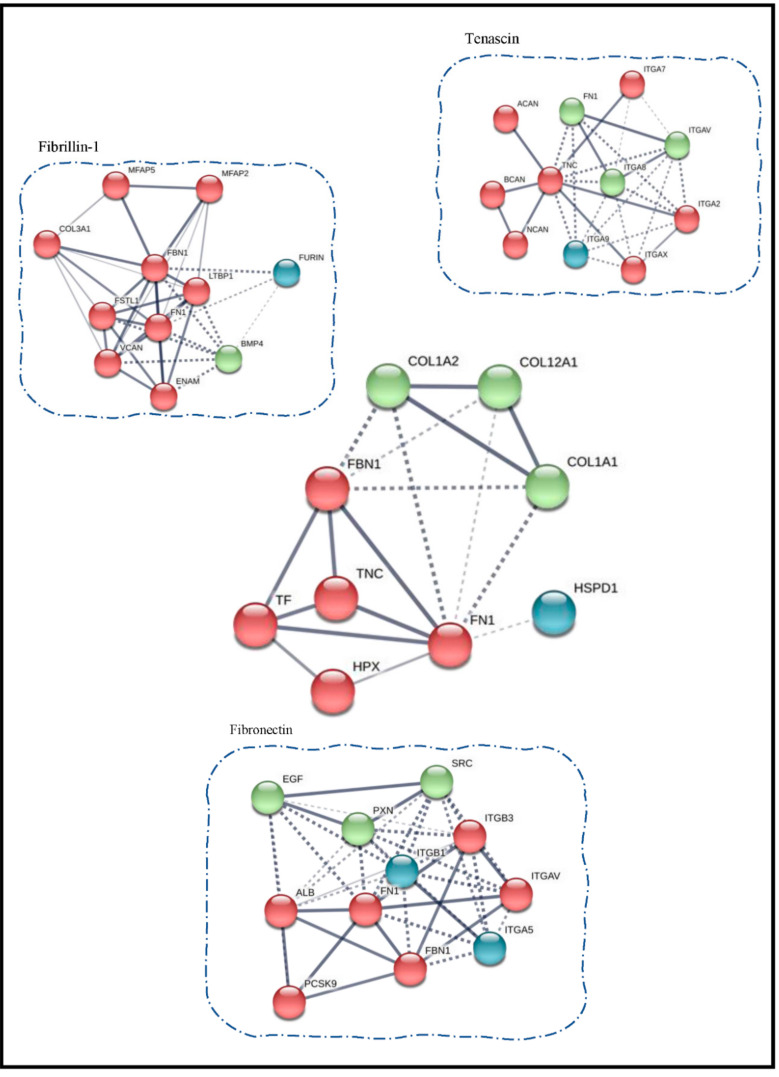
Clustering of proteins using STRING is shown. The center cluster is from the common proteins obtained in our study. The database was also searched for already known interacting partners of key proteins like Fibrillin-1, Fibronectin and Tenascin.

Furthermore, we also queried the STRING database through the list of common proteins that we obtained in our analysis (Figure 6). The central portion of Figure 6 highlights two small clusters that were obtained after removing all the secretory proteins from the list. We also observed some important proteins that have overlapping connections among other clusters are Fibronectin (FN1), Fibrillin 1 (FBN1), and Tenascin (TNC). 

Additionally, we searched the STRING database for the available information from published literature on interacting partners of these key proteins that we found to be overlapping and connected with every other cluster for instance the interacting partners of Tenascin (TNC) include Fibronectin (FN1), Aggrecan core protein (ACAN), both of which are present in our list of identified proteins. Similarly, most of the interacting partners of Fibronectin (FN1) and Fibrillin-1 (FBN1) were also present in our extracts. This backtracking validates our MS-based identification also.

## 4. Conclusions

Our study highlights the compositional differences between the four major types of human teeth for inorganic and organic constituents. Since the key inorganic component is hydroxyapatite (HA), which is common in all teeth types, the presence of several trace elements was investigated in this current study. These trace elements can substitute various ions including Ca^2+^ and PO4^2-^ and may regulate the apatite crystal formation. We observed variations in different trace elements concentration within different teeth types, for instance, a selective abundance of Zinc (Zn) and Aluminum (Al) was shown in incisor teeth whereas manganese (Mn) and copper (Cu) concentrations were found elevated in the premolar teeth. Such observations have never been made in the past. Though further studies are needed to draw strong conclusions, our study may be instrumental in initiating the argument for purposeful advancement in this direction.

For analyzing the organic structural components, we have performed label-free quantification of protein extracts from four subtypes of teeth. We have found qualitative and quantitative differences. Some of the key proteins involved in tooth development have shown varying abundance levels among diverse teeth types. The list includes Serotransferrin, Fibronectin, Fibrillin-1, Versican core protein, Tenascin, and Vitronectin to name a few. Additionally, we identified and represented some known interactions within the STRING database which corroborated well with structural proteins we identified in our MS-based experiments. Upon narrowing down of clustering results, Fibrillin-1, Fibronectin, and Tenascin were found to be the most interactive proteins and are responsible for interconnecting many clusters. We acknowledge the technical limitations associated with the availability of biomaterial like teeth to qualify our findings on rigorous statistical standards. Even though, this report should work as a preliminary reference to future compositional relevant studies.

## Figures and Tables

**Table 1:** List of key proteins that are common among different teeth types. C: canine; I: incisor; M: molar; P: premolar.

Teeth Type	C:I:M:P	C:M:I	C:I:P	M:I:P	C:I	C:M	P:I
Key proteins common in different teeth types	Collagen alpha-1(Q99715), Fibrillin-1 (P35555), Fibronectin (P02751), Collagen alpha-2(P08123), Hemopexin (P02790), Superoxide dismutase [Cu-Zn] (P00441), Serotransferrin (P02787), 60 kDa heat shock protein (P10809), Collagen alpha-1(P02452), Tenascin (P24821)	Alpha-amylase 2B precursor (P19961), Resistin precursor (Q9HD89), Alpha-amylase 1 precursor (P04745)	Versican core protein precursor (P13611), Vitronectin precursor (P04004), Prolow density lipoprotein receptor-related protein(Q07954), Thrombospondin-1 precursor (P07996), Collagen alpha-1(I) chain precursor (P02452), Filamin-A (P21333), Plasminogen precursor (P00747), Neutrophil defensin 3 precursor (P59666), Lactotransferrin precursor (P02788), Alpha-1-acid glycoprotein 1 precursor (P02763), Collagen alpha-1(XXVIII) chain precursor (Q2UY09), Neutrophil defensin 1 precursor (P59665), Tenascin-N precursor (Q9UQP3), Basement membrane-specific heparan sulfate (P98160), Prothrombin precursor (P00734)	Various subtypes of Keratins (P02538, P13647, P04259, O95678, P48668), Protein S100 (P06702), Laminin (P11047), Galectin-3-binding protein (Q08380)	Kininogen-1 precursor (P01042), Polyubiquitin-B precursor (P0CG47), Ubiquitin protein (P62987), Dickkopf-related protein 3 precursor (Q9UBP4)	Nucleolar RNA helicase 2 (Q9NR30)	Collagen alpha-2 (P08572), Aggrecan core protein(P16112), Filamin-B (O75369), Thrombospondin (Q6ZMP0), Zinc-alpha-2-glycoprotein (P25311)

## Supplementary Materials

The following are available online at https://www.mdpi.com/2218-273X/10/11/1540/s1, Figure S1: Gene ontology enrichment in proteins from different teeth types, Table S1: Complete list of identified proteins in human tooth extracts, Table S2: Complete list of proteins shown in the Venn diagram, Table S3 (Tables 1–6): Complete list of differentially expressed proteins between all tooth types. The mass spectrometry proteomics data have been deposited to the ProteomeXchange Consortium via the PRIDE partner repository with the dataset identifier PXD022223.
